# Application of Convolutional Neural Network for Fingerprint-Based Prediction of Gender, Finger Position, and Height

**DOI:** 10.3390/e24040475

**Published:** 2022-03-29

**Authors:** Chung-Ting Hsiao, Chun-Yi Lin, Po-Shan Wang, Yu-Te Wu

**Affiliations:** 1 Institute of Biophotonics and Brain Research Center, National Yang Ming Chiao Tung University, Taipei 11221, Taiwan; sbp681172@gmail.com (C.-T.H.); sifter7380@gmail.com (C.-Y.L.); 2 Forensic Science Center, New Taipei City Police Department, New Taipei City 22005, Taiwan; 3 Department of Neurology, Taipei Municipal Gan-Dau Hospital, Taipei 22360, Taiwan; b8001071@yahoo.com.tw

**Keywords:** fingerprint recognition, artificial neural network, image classification

## Abstract

Fingerprints are the most common personal identification feature and key evidence for crime scene investigators. The prediction of fingerprints features include gender, height range (tall or short), left or right hand, and finger position can effectively narrow down the list of suspects, increase the speed of comparison, and greatly improve the effectiveness of criminal investigations. In this study, we used three commonly used CNNs (VGG16, Inception-v3, and Resnet50) to perform biometric prediction on 1000 samples, and the results showed that VGG16 achieved the highest accuracy in identifying gender (79.2%), left- and right-hand fingerprints (94.4%), finger position (84.8%), and height range (69.8%, using the ring finger of male participants). In addition, we visualized the CNN classification basis by the Grad-CAM technique and compared the results with those predicted by experts and found that the CNN model outperformed experts in terms of classification accuracy and speed, and provided good reference for fingerprints that were difficult to determine manually.

## 1. Introduction

Fingerprints are the most common feature used in biometric technology for personal identification; hence, they are key evidence for crime scene investigators. In forensic science, unidentified fingerprints obtained from a crime scene are imported into a database for identification. The success rate for identification is determined by the size of the database, the quality of the fingerprints, and the fingerprint-matching algorithms that are used. Upon establishing a match from a database, investigators can identify a suspect and quickly solve a case. However, due to human rights challenges and legal restrictions, the data integrity of fingerprint databases in most countries is often poor and many fingerprints cannot be identified. Therefore, additional clues must be obtained from fingerprints during crime investigations. Biometric information, such as gender and height predicted using fingerprints [1,2,3], can also be effectively used to narrow a list of suspects; such information is particularly useful when combined with other investigative information such as surveillance images and witness testimony.

Early studies of fingerprints focused on analysing the spatial domain where spatial frequency features were calculated manually; these features include the number (or density) of minutia, the ratio of ridge thickness to valley thickness, ridge width, and fingerprint pattern. Most studies have indicated that female fingerprints contain more minutia in a given area [4]. The rapid growth of computing power has led to the gradual replacement of traditional ink-based fingerprint cards by digital electronic files. The combined use of image processing techniques (for extracting features from fingerprint images) and machine learning for fingerprint classification has become the primary research trend for gender prediction [5,6,7,8]. In 2012, Gnanasivam, et al. [9] proposed a fingerprint-based method for gender classification based on discrete wavelet transform and singular value decomposition; the method involves the use of the k-nearest neighbours’ classifier for gender classification and achieved overall accuracy rates of 91.67% and 84.69% for male (1980 samples) and female (1590 samples) fingerprints, respectively. In 2018, Shehu, et al. used the Resnet34 model [10] to analyse fingerprint images and achieved approximately 75%, 94%, and 77% accuracy in gender prediction, right- and left-handed prediction, and finger position prediction, respectively. In 2020, Kim, et al. used CNN models such as Alexnet, Resnet50, VGG16, and YOLO to classify left- and right-hand fingerprints, with Resnet50 achieving the highest accuracy rate of 96.8% [11]. In 2021, Rim, et al. compared CNN, Alexnet, VGG16, Yolo-v2, and Resnet50, with the Yolo-v2 model providing the highest accuracy for right and left hand, scratch, and fingers classification with 90.98%, 78.68%, and 66.55%, respectively [12].

Height is another crucial clue in criminal investigations. Although witnesses and victims often cannot accurately describe a suspect’s height, they can usually provide a height range. Given that height ranges can help investigators to quickly narrow a list of suspects, the present study also examined the height ranges (tall or short) predicted using fingerprint images.

**Figure 1:**
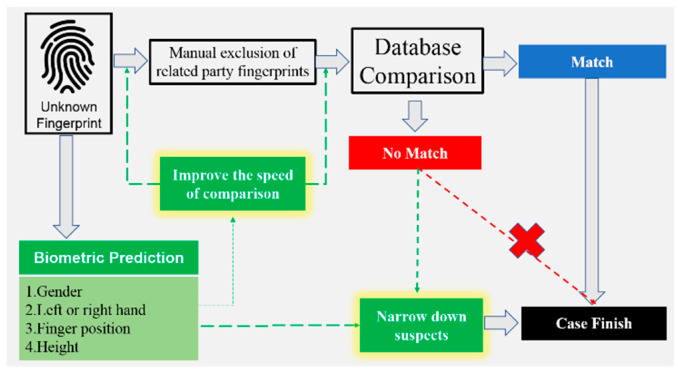
Study objectives.

In practice, crime scene investigators must collect not only suspicious fingerprints but also the fingerprint cards of related individuals (such as the victim and their relatives) to exclude them from the list of suspects. The exclusion of related individuals is usually performed manually. When the number of suspicious fingerprints and fingerprint cards (of related individuals) increases, the number of required comparisons increases dramatically. Therefore, the ability to determine the exact finger position from a fingerprint image can effectively improve the speed of comparison. For example, if a fingerprint collected from a crime scene is identified as the left index finger, the matching speed of comparisons for exclusion can be increased. When combined with other biometric characteristics (gender, left- or right-hand fingerprint, finger position, and height), fingerprint images can be even more useful in actual criminal investigations (Figure 1).

In summary, we know that the prediction of biometric features such as gender, height range (tall or short), left or right hand, and finger position can effectively narrow down the list of suspects, increase the speed of comparison, and greatly improve the effectiveness of criminal investigations. Since these four features have not been put together for a more complete and systematic analysis before, this study will use the recently popular CNN technique to perform the above analysis. In addition, we will invite experts in fingerprint identification to make the same predictions and visualize the CNN classification results using the Grad-CAM technique and ask experts to interpret the CNN classification results.

## 2. Materials and Methods

**Figure 2:**
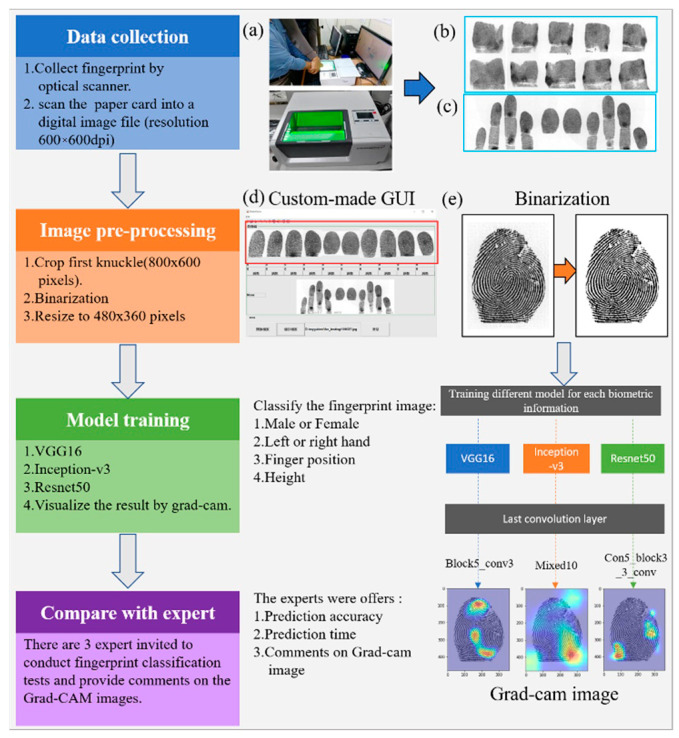
Study flowchart. (**a**) optical scanner for collecting fingerprint. (**b**) upper three-sided print area. (**c**) lower flat print area. (**d**) MATLAB graphical user interface to crop the first knuckle of each finger. (**e**) Binarization, converted the image to a binary colour scheme of black and white.

The present study used a sample of 1000 paper-based fingerprint cards (with an equal number of male and female fingerprint cards) provided by the New Taipei City Police Department. All personal identification data were removed from the cards. Figure 2 presents the study flowchart, which is described as follows.

### 2.1. Data Collection

The fingerprints were collected using a fingerprint optical scanner (Crossmatch L SCAN 500P, HID Global, Austin, TX, USA; Figure 2a), which scanned and converted the information on each paper-based fingerprint card into a digital image file (resolution 600 × 600 dpi; JPG file format). A sample of 1000 paper fingerprint cards (equal number of fingerprint cards for men and women) was collected as described previously. Their height distribution is shown in the Table 1.

### 2.2. Image Preprocessing

Each fingerprint card comprises an upper three-sided print area and a lower flat print area Figure 2b,c. Since flat prints are more commonly found at crime scenes, the present study focused on fingerprints from the lower flat print area. A custom-made MATLAB (MATLAB v. R2019b, The MathWorks Inc., Natick, MA, USA) graphical user interface Figure 2d was used to crop the first knuckle of each finger. The image size was 800 pixels × 600 pixels. To shorten the training time of CNN models, each image was scaled to 480 pixels × 360 pixels and converted to a binary colour scheme of black and white Figure 2e.

### 2.3. Establishment of Deep Learning Model

The present study used three commonly used deep learning models, namely VGG16, Inception-v3, and Resnet50 [13,14,15], which were implemented using Tensorflow 2.4.1 and Keras 2.4.0 in a Python 3.7.4 environment to automatically extract fingerprint features and perform classification. The model architecture is summarized in Supplementary File S1.

In addition, the four biometric targets of gender, left- and right-hand fingerprints, finger position, and height range (tall or short) were predicted using fingerprint images, and a total of 12 deep learning models were trained.

**Figure 3:**
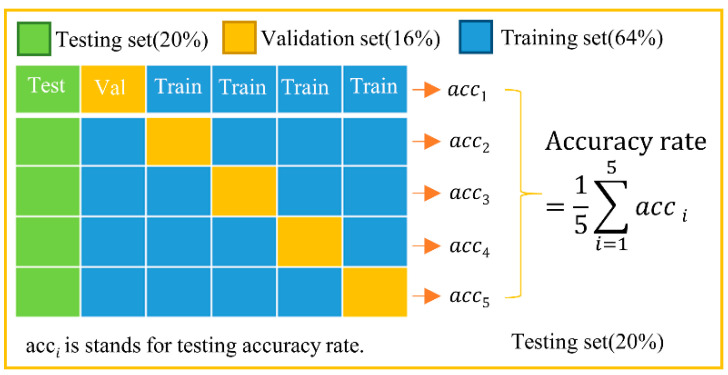
Five-fold cross-validation.

To avoid sampling bias for the test data set, a five-fold cross-validation was used in this study (as shown in Figure 3). A measure of 20% of the samples were used as the testing set and did not participate in the training. The remaining 80% of the samples were divided into training and validation set in a 4:1 ratio. In other words, the ratios of training dataset, validation dataset and testing dataset are 64%, 16%, and 20%, respectively. For model training, we used stochastic gradient descent as the optimiser for all models, a learning rate of 1 × 10^−5^ (excluding the prediction of finger position, for which the learning rate was 1 × 10^−4^), and a momentum of 0.9. The batch size was 10, the loss function was binary cross-entropy, and ten epochs of training iterations were performed. To prevent the model from overfitting during training, we added a 50% dropout to the fully connected layer and set the early stop to three epochs based on validation loss (i.e., training was halted if validation loss did not decrease for three consecutive epochs). In the subsequent sections, each reported testing accuracy value is the average value obtained from running the five validated models.

### 2.4. Grad-Cam Image Analysis and Experts’ Prediction

We used Gradient-weighted Class Activation Mapping (Grad-CAM) to identify image regions of interest for CNN models to perform fingerprint classification. Grad-CAM is a technique for visualising neural network decisions. Grad-CAM works by calculating the weights of multiple feature maps that correspond to multiple classes to localise the image area where a model is activated.

For the weights of each feature map, we first calculated the gradient of each pixel of the previous feature map (*A*) in the convolution layer by using back propagation and then summed the gradients of each feature map separately to derive the weights (*w*) of each feature map for multiple categories as follows:

(1) $$w_{k}^{c}=\underset{i}{\sum }\underset{j}{\sum }\frac{\partial Y^{c}}{\partial A_{ij}^{k}}\;$$

where *Y^c^* is the output of class c, *A^k^* is the *k*th feature map, and *w* is the weight corresponding to multiple different feature maps. The *Heatmap* is a linear combination of each weight (*w*) and the corresponding feature map (*A*), and the final result is the output using ReLU; a *Heatmap* is expressed represented as follows:

(2) $$Heatmap^{c}=ReLU\left(\underset{k}{\sum }w_{k}^{c}A^{k}\right)\;$$

This method is not restricted to global average pooling and is applicable to the fully connected layer architecture of a deep learning model [16,17].

In addition, three forensic investigators qualified to conduct fingerprint identification were invited to conduct fingerprint classification and provided feedback on the Grad-CAM images.

## 3. Results

### 3.1. Gender Prediction

In total, 4000 of 5000 fingerprint image samples from each gender were used for training, and 1000 samples from each gender were used for testing. Table 2 indicates that VGG16 and Resnet50 achieved similar classification rates (79.2% and 79.1%, respectively), whereas Inception-v3 achieved a lower rate of 77.1%. The ROC and PR curves of predictions were shown in Supplementary File S2.

### 3.2. Prediction of Left- or Right-Hand Fingerprints

In total, 10,000 fingerprint images with an equal number of images from left and right hands were used. Eight thousand and 2000 samples were used for training and testing, respectively. Table 3 indicates that VGG16, Resnet50, and Inception-v3 achieved classification rates of 94.4%, 93.8%, and 91.7%, respectively.

### 3.3. Prediction of Finger Position

To predict each finger position, 10,000 fingerprint images were divided into ten groups, with each group of 1000 samples corresponding to a specific finger position. Of the three CNNs, VGG16 achieved the highest overall accuracy of 84.8% for the prediction of finger position (for specific finger positions, the model achieved the highest accuracy of 96.5% for left thumb predictions and the lowest accuracy of 75% for left ring finger predictions). Table 4 presents the summary of the results.

We can see from Table 4 that the success rate of the thumb prediction is slightly higher than that of the other fingers, while the accuracy rate of the little finger is second, because the thumb and little finger are indeed more specific in shape than the other three fingers. The detailed predictions for each finger position are shown in Supplementary File S3.

### 3.4. Prediction of Height Range

We select males whose heights are larger than 180 cm or lower than 168 cm, and females whose heights are larger than 166 cm or lower than 155 cm and investigate whether fingerprints are useful for binary classification of heights (tall vs. short). This condition fits well with the scenario in practical work, where it is often difficult for investigators to determine the exact height of a suspect, but it is usually possible to obtain a description of the suspect as being particularly tall or particularly short.

Fingerprint images of the 100 tallest (180 to 195 cm) and 100 shortest (154 to 168 cm) male individuals were selected and divided into two groups (which are high and short group, and each group has 1000 fingerprints). Three deep learning models were used for height prediction. For all fingers, the three models achieved similar accuracy rates, specifically 63.9%, 63.8%, and 63.7% for VGG16, Inception-v3, and Resnet50, respectively.

With respect to specific finger positions, VGG16 achieved the highest accuracy rate of 69.8% for classifying ring fingers. Table 5 present the male height prediction results. The detailed predictions for height from specific finger position were shown in Supplementary File S4.

Fingerprint images of the 100 tallest (166 to 179 cm) and 100 shortest (137 to 155 cm) female individuals were selected and divided into two groups. For all fingers, VGG16 and Resnet50 achieved higher accuracy rates (62.5% and 62.8%, respectively) relative to Inception-v3 (60.5%).

With respect to specific finger positions, Inception-v3 achieved the highest accuracy rate of 68.8% for classifying little fingers. Table 6 present the female height prediction results.

High accuracy was not achieved in predicting the binary classification of height (tall vs. short) and would be even lower if we considered adding medium height or more categories. This is not applicable to practical work currently and requires more studies in the future.

### 3.5. Expert Prediction and Grad-CAM Image Analysis Results

Three criminal scene investigators (i.e., experts) who were qualified to conduct fingerprint identification were invited to conduct fingerprint classification and provide feedback on the Grad-CAM images. The investigators, who are referred to as A, B, and C, had 5, 10, and 11 years of relevant work experience, respectively.

Twenty fingerprints with an equal number of male and female fingerprints were randomly selected from the test data and submitted to the experts for gender prediction. The experts achieved an overall accuracy rate of 65% and spent an average of 73.3 s on each image (Table 7).

**Figure 4:**
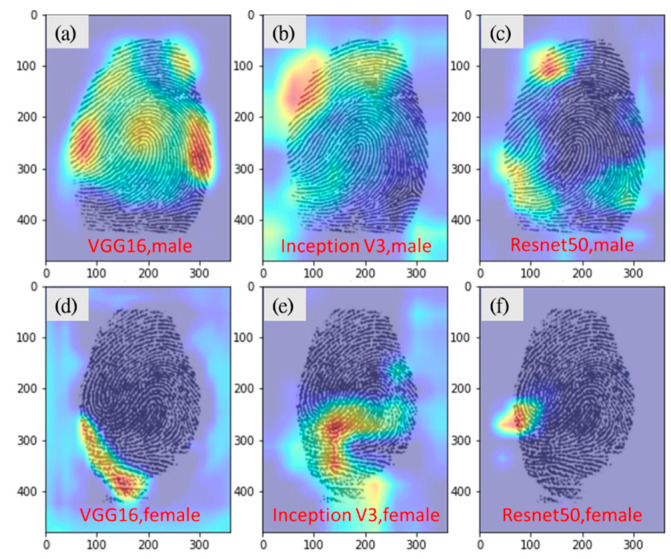
Grad-CAM images of correct gender predictions. (**a**) VGG16, correct prediction for male. (**b**) Inception-v3, correct prediction for male. (**c**) Resnet50, correct prediction for male. (**d**)VGG16, correct prediction for female. (**e**) Inception-v3, correct prediction for female. (**f**) Resnet50, correct prediction for female.

In addition, the three experts analysed the Grad-CAM images and revealed that the density of fingerprint ridges was a key feature learned by the deep learning model for classification. The ridges of male and female fingerprints are less and more densely packed, respectively (Figure 4). The model used features similar to those used by humans to make predictions during visual inspections; however, VGG16 was more accurate (79.2%) than the three experts.

**Figure 5:**
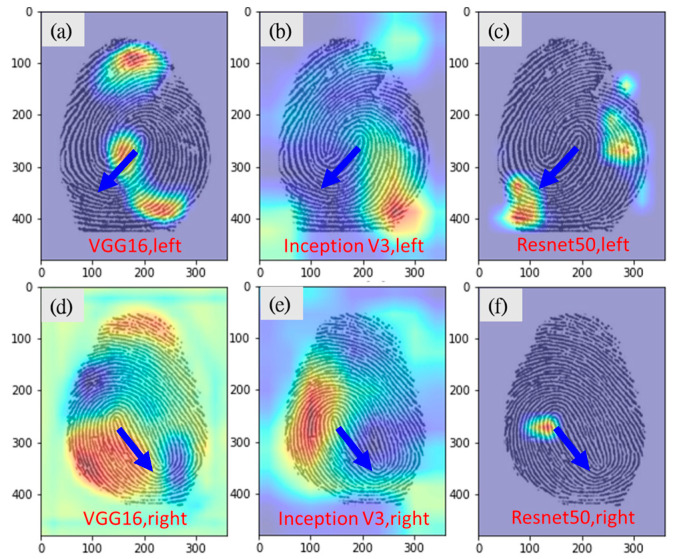
Grad-CAM images of correct left- and right-hand fingerprint predictions (the blue arrow show the direction of the skip). (**a**) VGG16, correct prediction for left. (**b**) Inception-v3, correct prediction for left. (**c**) Resnet50, correct prediction for left. (**d**) VGG16, correct prediction for right. (**e**) Inception-v3, correct prediction for right. (**f**) Resnet50, correct prediction for right.

Twenty fingerprints, with an equal number of left- and right-hand fingerprints were randomly selected from the testing data set and submitted to the three experts who then predicted whether each fingerprint was a left- or right-hand fingerprint. The experts achieved an overall accuracy rate of 83.3% and required an average of 72.3 s for each image (Table 8). They also analysed the Grad-CAM images and discovered that fingerprint pattern flow is a key feature learned by the deep learning model for classification. Left- and right-hand fingerprints have ridges that mostly rotate clockwise and counter clockwise, respectively (Figure 5). The model used features similar to those used by humans to make predictions; however, VGG16 was much more accurate (94.4%) than the three experts.

It is worth mentioning that experts can easily determine the left hand and right hand from the loop pattern fingerprint by the direction of the skip (towards the left is the left hand, as shown by blue arrow in Figure 5). However, other types of fingerprints (especially whorl patterns) are difficult to determine.

**Figure 6:**
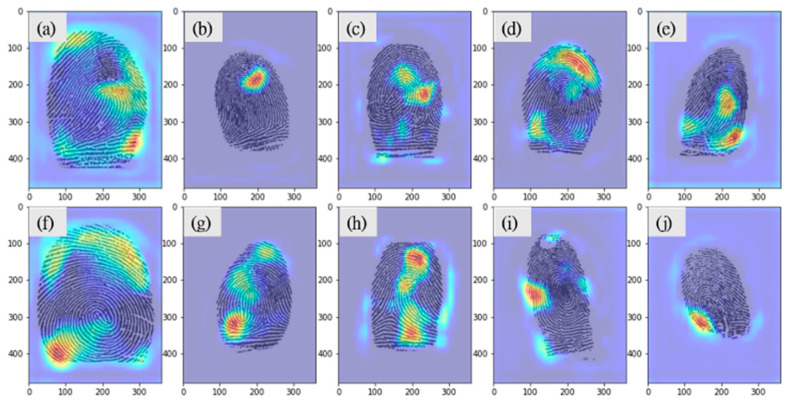
Grad-CAM images of correct finger position predictions by VGG16. (**a**) Left thumb; (**b**) left index finger; (**c**) left middle finger; (**d**) left ring finger; (**e**) left little finger; (**f**) right thumb finger; (**g**) right index finger; (**h**) right middle finger; (**i**) right ring finger; (**j**) right little finger.

Twenty fingerprints (two for each finger position) were randomly selected from the test data and submitted to the experts for finger position prediction. They achieved an overall accuracy rate of only 40% and required an average of 174 s for each image (Table 9). The three experts reported that the thumb and little fingers were easier to identify because of their distinctive appearances and that the other fingers were difficult to identify because they had no distinctive features. The three experts examined the Grad-CAM images (Figure 6) and did not identify any features for determining finger positions with respect to index, middle, and ring fingers.

**Figure 7:**
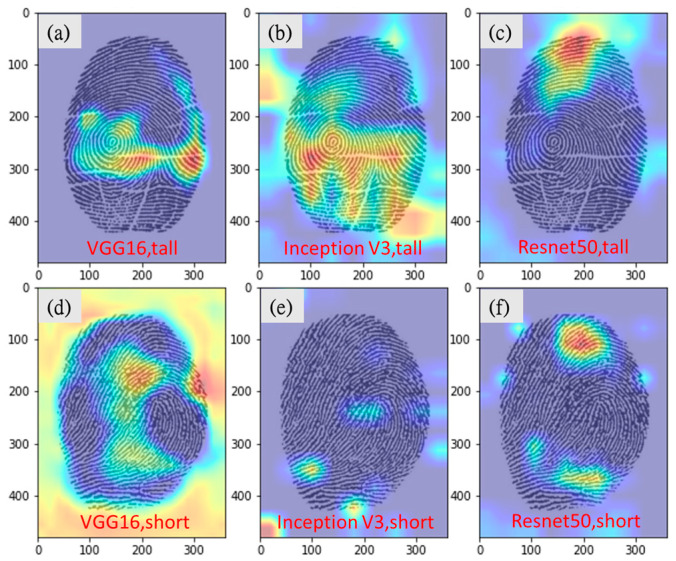
Grad-CAM images of correct male height predictions. (**a**) VGG16, correct prediction for tall. (**b**) Inception-v3, correct prediction for tall. (**c**) Resnet50, correct prediction for tall. (**d**) VGG16, correct prediction for short. (**e**) Inception-v3, correct prediction for short. (**f**) Resnet50, correct prediction for short.

Twenty fingerprints were randomly selected from the test data and submitted to the experts for height range (tall or short) prediction. The predictions were made based on fingerprint size and ridge width. For male and female height range predictions, they achieved overall accuracy rates of 56.7% and 51.7%, respectively, and required an average of 60.7 and 56.3 s, respectively (Table 10). The three experts could not identify any notable features in the Grad-CAM images for aiding height range prediction (Figure 7).

## 4. Discussion

The accuracy of this study in predicting features such as gender, left- or right-hand, and finger position is comparable with that obtained by Shehu et al. using the Resnet34 model [10]. However, we also found that VGG16 achieved better results in terms of accuracy compared to inception v3 and Resnet50.

From earlier studies, we learned that the ridge density of fingerprint patterns is an important basis for gender determination [4]. Such a method requires tedious manual calculations for minutia, the ratio of ridge thickness to valley thickness, or ridge width. Based on the Grad-Cam images which are consistent with the features adopted by fingerprint identification experts, we found that the CNN model also makes gender judgment based on the ridge density of fingerprint patterns, and the accuracy is higher (79.2% from VGG16 versus 65%) and faster than human prediction, suggesting that we can use the CNN model to build a fast gender prediction, or highlight sensitive regions to facilitate manual judgment.

**Figure 8:**
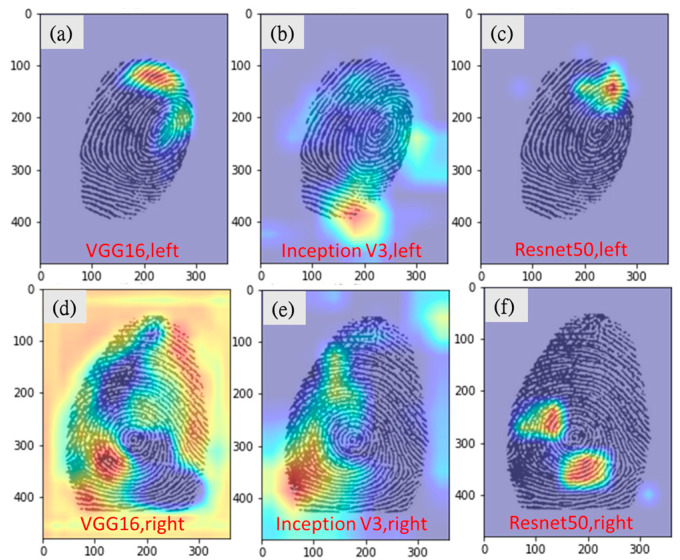
Grad-CAM images of correct left- and right-hand predictions for whorl-pattern fingerprints. (**a**) VGG16, correct prediction for left. (**b**) Inception-v3, correct prediction for left. (**c**) Resnet50, correct prediction for left. (**d**) VGG16, correct prediction for right. (**e**) Inception-v3, correct prediction for right. (**f**) Resnet50, correct prediction for right.

Experts usually classify the left- and right-hand fingerprints according to the flow direction of the lines (most of the left and right hand fingerprints are rotated in clockwise and counter clockwise directions, respectively). In this study, the Grad-cam heat map indicated that the CNN model also learns similar features to classify left and right hand. The VGG16 model predicted the left or right hand with an accuracy of 94.4%, which is far superior to the manual prediction of 83.3%. This is mainly because even in some fingerprints where the human eye has difficulty in distinguishing the direction of flow (usually whorls, as shown in Figure 8), the CNN model has better sensitivity than the human eye to identify the sensitive areas for correct prediction.

In this study, the accuracy of the experts in predicting finger positions was only 40%, which indicate that the experts do not have a reliable feature on judging the finger positions. In contrast, from the prediction results of the CNN model, the accuracy of the thumb and little finger was higher than the other three fingers (with the thumb reaching more than 95%). The main reasons could be because of two obvious features, namely, shape and size, of the thumb and little finger. In addition, the CNN model should have found additional hidden features so that it achieved more than 80% accuracy in the index, middle and ring fingers, although their shapes and sizes are similar. Nevertheless, neither the Gram-cam heat map nor expert analysis was able to reveal such hidden features. In future studies, we will exclude the effect of shape and size to further investigate the features learned by CNNs for classification of finger positions.

To date, few studies have reported on the correlation between fingerprints and height. As described earlier, we conduct a binary classification of height (tall vs. short), a condition that fits well with the actual working scenario. However, from the results of the study, neither expert prediction (with an accuracy of about 50%) nor deep learning inference (with an accuracy of about 60%) achieved high accuracy in predicting binary classification of heights (tall vs. short), and if we consider adding medium height or more categories, the accuracy will be even lower. Although the accuracy of the CNN model is higher than “expert guesses”, it does not seem to be applicable to practical work for now. We will collect more fingerprint and height samples in the future to see if we can further improve the accuracy or confirm that there is no direct correlation between height and fingerprint.

For height range predictions (tall or short), neither the expert predictions (accuracy rate of approximately 50%) nor the deep learning models (accuracy rate of approximately 60%) achieved high accuracy rates. In addition, we performed linear regression for height prediction. The height distribution of male ranges from 154 to 195 cm and that of female from 137 to 179 cm; 80% and 20% of the samples were used for training and testing, respectively. Results indicate that the mean absolute error (MAE) for male individuals was 6.62 cm with a maximum absolute error of 27 cm in predicting individuals measuring 190 to 200 cm; 47% of the samples had an MAE of 5 cm or less. The MAEs were larger for samples associated with individuals measuring 150–160 cm, 180–190 cm, and 190–200 cm; this could be attributed the smaller training sample size for these height ranges relative to the height ranges of 160–170 cm and 170–180 cm. The MAE was 5.62 cm for female individuals with an MAE of 11.5 cm in predicting individuals measuring 140–150 cm; 48% of the samples had an MAE of 5 cm or less. The results for male and female individuals indicated that MAE decreased substantially when the number of testing samples was increased. In future studies, we will collect more samples such that the sample size for each height range is similar for both males and female individuals, which will improve the overall accuracy of height prediction.

## 5. Conclusions

In criminal investigation cases, quickly determining the gender or height of a suspect can effectively help narrow down the suspect. Accurate prediction of left and right hand or finger position can effectively improve the speed of comparison and can also be used as a tool for criminal investigators to exclude fingerprints of victims and their relatives. In this study, we conducted complete comparison and analysis of biometric features such as gender, left and right hand, finger position and range of height by CNN model, and the CNN model outperformed human experts in terms of accuracy and speed. In addition, after visualizing the classification basis of CNN by Grad-CAM technique and combining with expert opinions, we found that the CNN model can provide reliable analysis results for fingerprints that are difficult to be judged manually, which is very helpful to assist manual research and evaluation.

## Figures and Tables

**Table 1:** Height distribution of samples.

Male		Female	
Height	Counts	Height	Counts
140–150	0	140–150	11
150–160	3	150–160	173
160–170	100	160–170	263
170–180	289	170–180	52
180–190	102	180–190	1
190–200	6	190–200	0

**Table 2:** Gender prediction testing results obtained from five validated models of VGG16, Inception-v3, and Resnet50.

Model	Male		Female		Accuracy (Mean ± STD)
	Correct	Incorrect	Correct	Incorrect	
VGG16	766.6	233.4	817.6	182.4	79.2 ± 1.2%
Inception-v3	774.8	225.2	767	233	77.1 ± 0.9%
Resnet50	793	207	789.6	210.4	79.1 ± 0.8%

**Table 3:** Left- and right-hand prediction testing results obtained from the validated models of VGG16, Inception-v3, and Resnet50.

Model	Left		Right		Accuracy (Mean ± STD)
	Correct	Incorrect	Correct	Incorrect	
VGG16	950.4	49.6	937.8	62.2	94.4 ± 0.5%
Inception-v3	917.6	82.4	916.6	83.4	91.7 ± 0.5%
Resnet50	928.8	71.2	944.6	55.4	93.7 ± 0.5%

**Table 4:** Finger position prediction testing results obtained from the validated models of VGG16, Inception-v3, and Resnet50.

Model	Left/Right	Thumb	Index	Middle	Ring	Little	Average Accuracy
VGG16	Left	96.5 ± 0.7%	82.3 ± 3.0%	79.3 ± 2.2%	75.0 ± 3.0%	86.0 ± 2.9%	84.8%
	Right	95.5 ± 2.4%	87.9 ± 3.8%	81.8 ± 3.8%	75.2 ± 3.4%	88.4 ± 2.3%	
Inception-v3	Left	94.2 ± 2.0%	69.9 ± 4.1%	67.9 ± 5.0%	71.9 ± 3.7%	80.5 ± 3.4%	77.9%
	Right	93.6 ± 1.7%	76.7 ± 5.4%	71.8 ± 4.5%	72.5 ± 5.0%	80.1 ± 4.5%	
Resnet50	Left	95.6 ± 1.3%	80.9 ± 4.4%	79.1 ± 2.8%	70.8 ± 4.3%	82.7 ± 3.3%	83.9%
	Right	96.3 ± 3.0%	82.9 ± 3.6%	77.4 ± 5.6%	79.5 ± 2.9%	84.2 ± 2.2%	

**Table 5:** Male height prediction testing results obtained from five validated models.

Model	Tall		Short		Accuracy (Mean ± STD)
	Correct	Incorrect	Correct	Incorrect	
VGG16	131.8	68.2	123.8	76.2	63.9 ± 4.1%
Inception-v3	124.2	75.8	131	69	63.8 ± 3.1%
Resnet50	113	87	141.8	58.2	63.7 ± 2.2%

**Table 6:** Female height prediction testing results obtained from five validated models.

Model	Tall		Short		Accuracy (Mean ± STD)
	Correct	Incorrect	Correct	Incorrect	
VGG16	123.4	76.6	126.4	73.6	62.5 ± 1.9%
Inception-v3	146.8	53.2	95.2	104.8	60.5 ± 2.3%
Resnet50	98.4	101.6	152.8	47.2	62.8 ± 3.6%

**Table 7:** Results of experts’ gender predictions.

Expert	Male		Female		Accuracy	Time
	Correct	Incorrect	Correct	Incorrect		
A	6	4	9	1	75.0%	57 s
B	6	4	6	4	60.0%	85 s
C	6	4	6	4	60.0%	78 s
Average	18	12	21	9	65.0%	73.3 s

**Table 8:** Results of experts’ left- and right-hand fingerprint predictions.

Expert	Left		Right		Accuracy	Time
	Correct	Incorrect	Correct	Incorrect		
A	8	2	9	1	85.0%	57 s
B	8	2	9	1	85.0%	62 s
C	7	3	9	1	80.0%	98 s
Average	23	7	27	3	83.3%	72.3 s

**Table 9:** Experts’ finger position predictions.

Expert	Correct	Incorrect	Accuracy	Time
A	9	11	45.0%	169 s
B	6	14	30.0%	131 s
C	9	11	45.0%	222 s
Average	24	36	40.0%	174 s

**Table 10:** Experts’ male and female height range predictions.

Male					
Model	Tall		Short		Accuracy (Mean ± STD)
	Correct	Incorrect	Correct	Incorrect	
VGG16	131.8	68.2	123.8	76.2	63.9 ± 4.1%
Inception V3	124.2	75.8	131	69	63.8 ± 3.1%
Resnet50	113	87	141.8	58.2	63.7 ± 2.2%
**Female**					
**Model**	**Tall**		**Short**		**Accuracy** **(Mean ± STD)**
	**Correct**	**Incorrect**	**Correct**	**Incorrect**	
VGG16	123.4	76.6	126.4	73.6	62.5 ± 1.9%
Inception V3	146.8	53.2	95.2	104.8	60.5 ± 2.3%
Resnet50	98.4	101.6	152.8	47.2	62.8 ± 3.6%

## Supplementary Materials

The following supporting information can be downloaded at: https://www.mdpi.com/article/10.3390/e24040475/s1, Supplementary File S1: The structure of deep learning model; Supplementary File S2: The ROC and PR curves of predictions; Supplementary File S3: The predictions of each finger position; Supplementary File S4: The predictions of height from specific finger position.

## References

[1] Karki R., Singh P. (2014). Gender determination from fingerprints. J. Univ. Coll. Med. Sci..

[2] Falsetti A.B. (1995). Sex Assessment from Metacarpals of the Human Hand. J. Forensic Sci..

[3] Badawi A.M., Mahfouz M., Tadross R., Jantz R. (2006). Fingerprint-Based Gender Classification. IPCV.

[4] Ramanjit K., Garg R.K. (2011). Determination of Gender Differences from Fingerprint Ridge Density in Two Northern Indian Populations. Probl. Forensic Sci..

[5] Tom R.J., Arulkumaran T., Scholar M.E. (2013). Fingerprint based gender classification using 2D discrete wavelet transforms and principal component analysis. Int. J. Eng. Trends Technol..

[6] Gornale S.S., Geetha C.D., Kruthi R. (2013). Analysis of fingerprint image for gender classification using spatial and frequency domain analysis. Am. Int. J. Res. Sci. Technol. Eng. Math..

[7] Kaur R., Mazumdar S.G. (2012). Mazumdar, and Technology, Fingerprint based gender identification using frequency domain analysis. Int. J. Adv. Eng. Technol..

[8] Rajesh D.G., Punithavalli M. (2014). An efficient fingerprint based gender classification system using dominant un-decimated wavelet coefficients. Res. J. Appl. Sci. Eng. Technol..

[9] Gnanasivam P., Muttan D.S. (2012). Fingerprint gender classification using wavelet transform and singular value decomposition. arXiv.

[10] Shehu Y.I., Ruiz-Garcia A., Palade V., James A. Detailed identification of fingerprints using convolutional neural networks. Proceedings of the 2018 17th IEEE International Conference on Machine Learning and Applications (ICMLA).

[11] Kim J., Beanbonyka R., Sung N.-J., Hong M. (2020). Left or Right Hand Classification from Fingerprint Images Using a Deep Neural Network. Comput. Mater. Contin..

[12] Rim B., Kim J., Hong M. (2021). Fingerprint classification using deep learning approach. Multimedia Tools Appl..

[13] Simonyan K., Zisserman A. (2014). Very deep convolutional networks for large-scale image recognition. arXiv.

[14] Szegedy C., Liu W., Jia Y., Sermanet P., Reed S., Anguelov D., Erhan D., Vanhoucke V., Rabinovich A. Going deeper with convolutions. Proceedings of the IEEE Conference on Computer Vision and Pattern Recognition (CVPR).

[15] He K., Zhang X., Ren S., Sun J. Deep residual learning for image recognition. Proceedings of the IEEE Conference on Computer Vision and Pattern Recognition.

[16] Selvaraju R.R., Cogswell M., Das A., Vedantam R., Parikh D., Batra D. Grad-cam: Visual explanations from deep networks via gradient-based localization. Proceedings of the IEEE International Conference on Computer Vision.

[17] Chattopadhay A., Sarkar A., Howlader P., Balasubramanian V.N. Grad-cam++: Generalized gradient-based visual explanations for deep convolutional networks. Proceedings of the 2018 IEEE Winter Conference on Applications of Computer Vision (WACV).

