# Supplementary material for: Application of Convolutional Neural Network for Fingerprint-Based Prediction of Gender, Finger Position, and Height
Source: Entropy (Basel). 2022 Mar 29;24(4):475. doi: 10.3390/e24040475 (PMC9029985; doi:10.3390/e24040475)
Supplement: Supplementary file 1 [file entropy-24-00475-s001.zip › Supplementary File S1.pdf]

Supplementary File S1. The structure of deep learning model

Table S1. Structure of VGG16

Layers	Filter size/stride	Input Size
InputLayer	480,360,3	
Conv.	[3x3,64]/1	480,360,3
Conv.	[3x3,64]/1	480,360,64
Maxoooling	[2x2]/2	480,360,64
Conv.	[3x3,128]/1	240,180,64
Conv.	[3x3,128]/1	240,180,128
Maxoooling	[2x2]/2	240,180,128
Conv.	[3x3,256]/1	120,90,128
Conv.	[3x3,256]/1	120,90,256
Conv.	[3x3,256]/1	120,90,256
Maxoooling	[2x2]/2	120,90,256
Conv.	[3x3,512]/1	60,45,256
Conv.	[3x3,512]/1	60,45,512
Conv.	[3x3,512]/1	60,45,512
Maxoooling	[2x2]/2	60,45,512
Conv.	3*3(512)/1	30,22,512
Conv.	3*3(512)/1	30,22,512
Conv.	3*3(512)/1	30,22,512
Maxoooling	[2x2]/2	30,22,512
FC	Flatten=84,480	
Dense	256， relu	
Dropout	0.5	
Softmax		

Table S3. Structure of Resnet50

Layers	Filter size/stride	Input Size
InputLayer	480,360,3	
Conv.	[3x3,32]/2	480,360,3
Conv.	[3x3,32]/1	239,179,32
Conv. padded	[3x3,64]/1	237,177,32
Pool	[3x3,64]/2	237,177,64
Conv.	[1x1,80]/1	118,88,64
Conv.	[3x3,192]/1	118,88,80
Pool	[3x3,192]/2	116,86,192
3*Inception	As Fig.3(a)	57,42,192
1*Inception	As Fig.3(a-1)	28,20,768
4*Inception	As Fig.3(b)	28,20,768
1*Inception	As Fig.3(b-1)	28,20,768
2*Inception	As Fig.3(c)	13,9,1280
FC	Flatten=239,616	
Dense	256， relu	
Dropout	0.5	
Softmax		

Table S2. Structure of Inception-v3

Layers	Filter size/stride	Output
InputLayer	480,360,3	
Conv.	[7x7,64]/2	480,360,3
Maxpooling	[3x3]/2	240,180,64
Conv.	[1x1,64]<br>[3x3,64] x3<br>[1x1,256]	120,90,64
Conv.	[1x1,128]<br>[3x3,128] x4<br>[1x1,512]	120,90,256
Conv.	[1x1,256]<br>[3x3,256] x6<br>[1x1,1024]	64,45,512
Conv.	[1x1,512]<br>[3x3,512] x3<br>[1x1,2048]	30,23,1024
FC	Flatten=368,640	
Dense	256，relu	
Dropout	0.5	
Softmax		

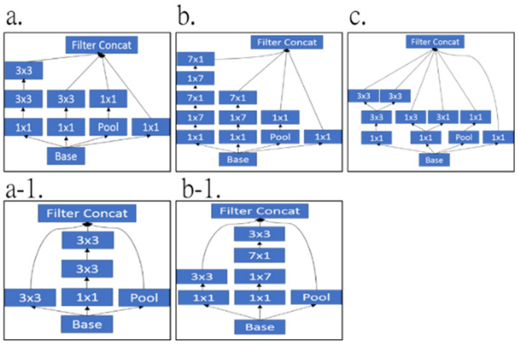

Figure S1. Inception unit
