# Supplementary material for: Application of Convolutional Neural Network for Fingerprint-Based Prediction of Gender, Finger Position, and Height
Source: Entropy (Basel). 2022 Mar 29;24(4):475. doi: 10.3390/e24040475 (PMC9029985; doi:10.3390/e24040475)
Supplement: Supplementary file 1 [file entropy-24-00475-s001.zip › Supplementary File S2.pdf]

Supplementary File S2. The ROC and PR curves of predictions

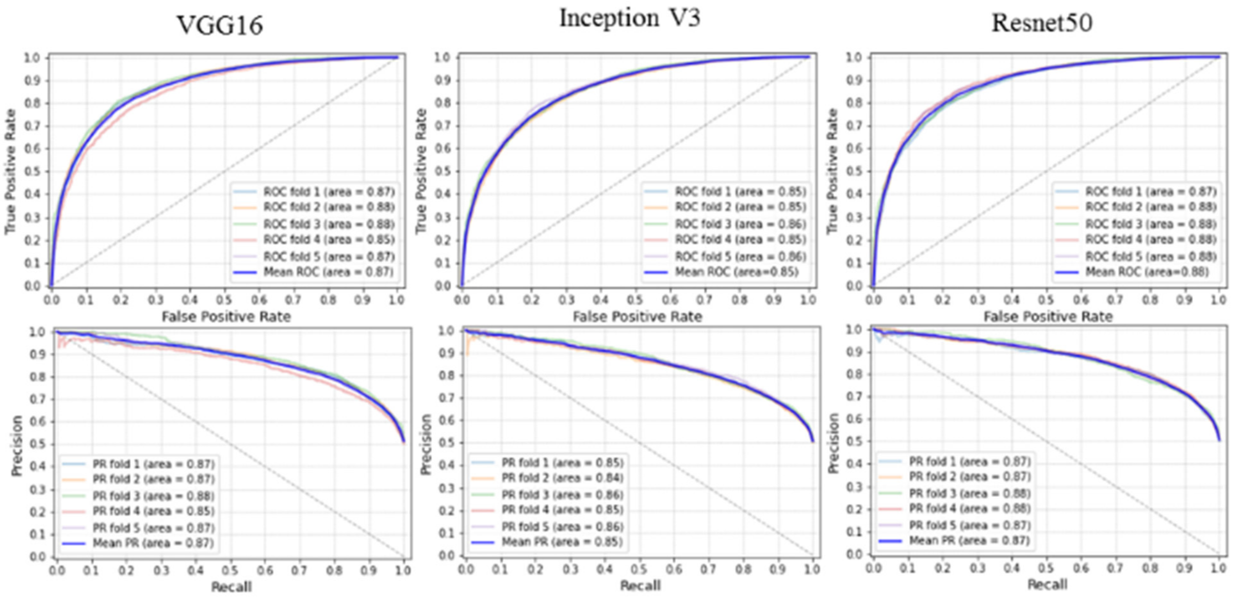

Figure S1. ROC and PR curves for gender prediction.

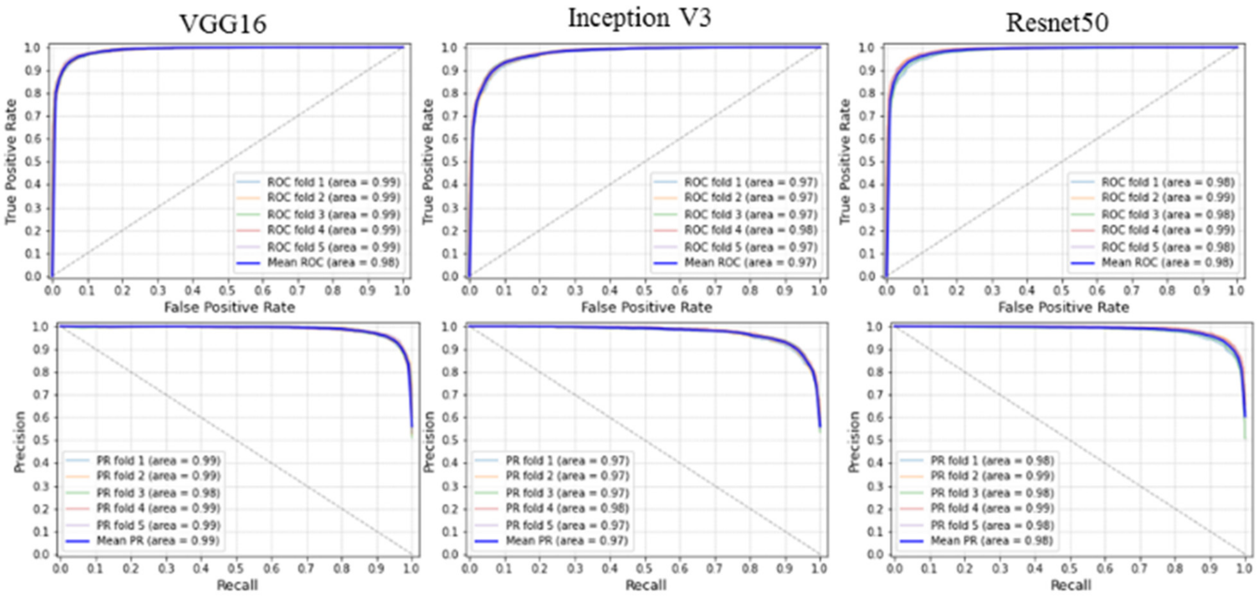

Figure S2. ROC and PR curves of left- and right-hand predictions.

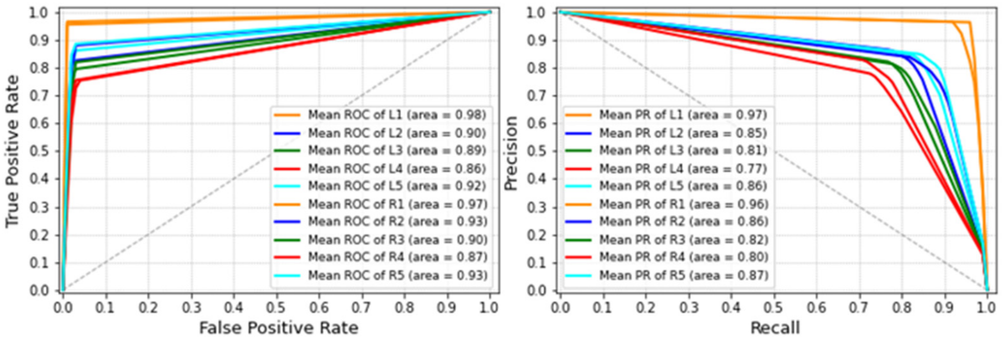

Figure S3. ROC and PR curves of finger position predictions by VGG16.

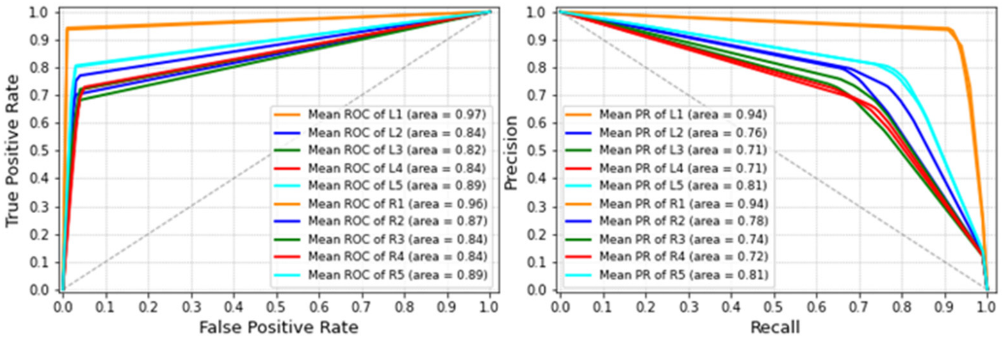

Figure S4. ROC and PR curves of finger position predictions by Inception-v3.

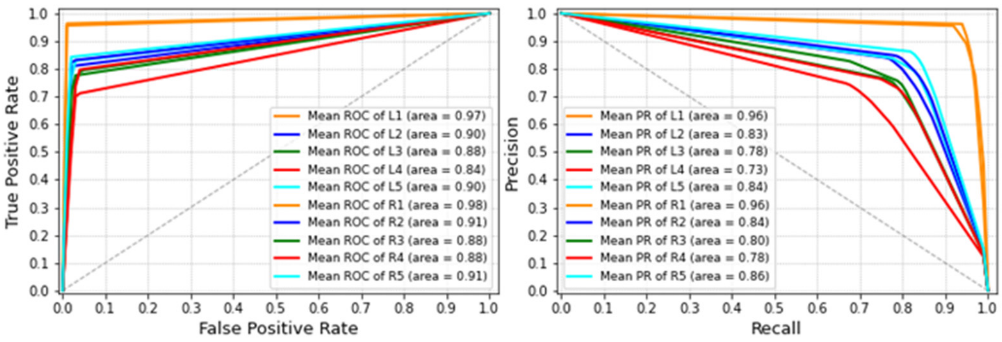

Figure S5. ROC and PR curves of finger position predictions by Resnet50.

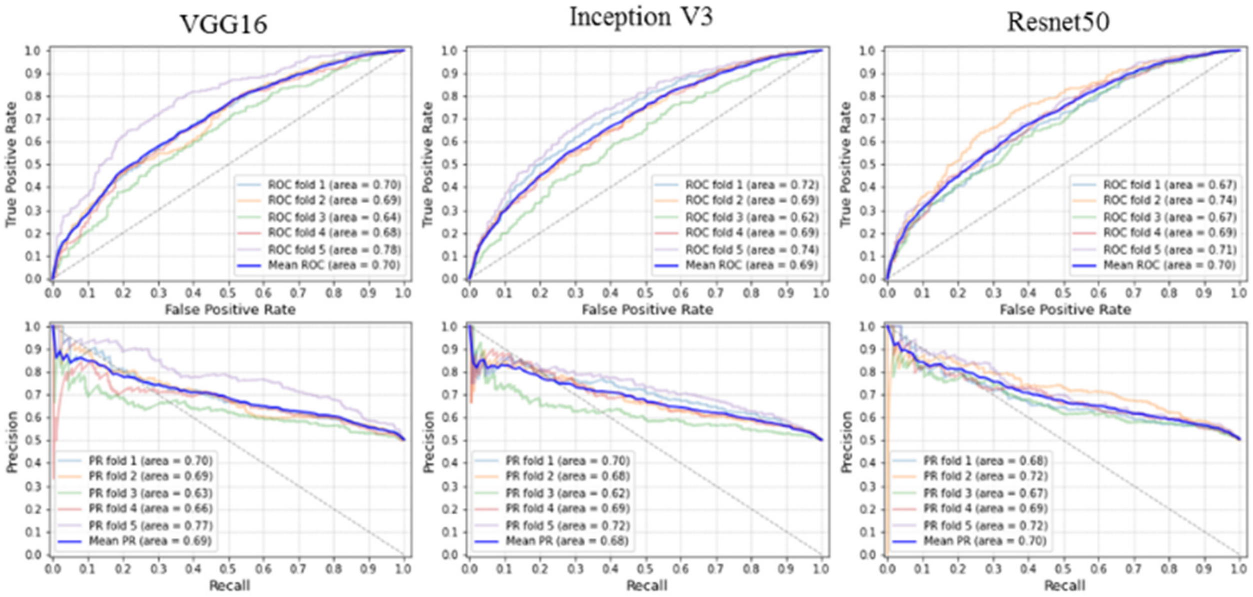

Figure S6. ROC and PR curves of male height predictions.

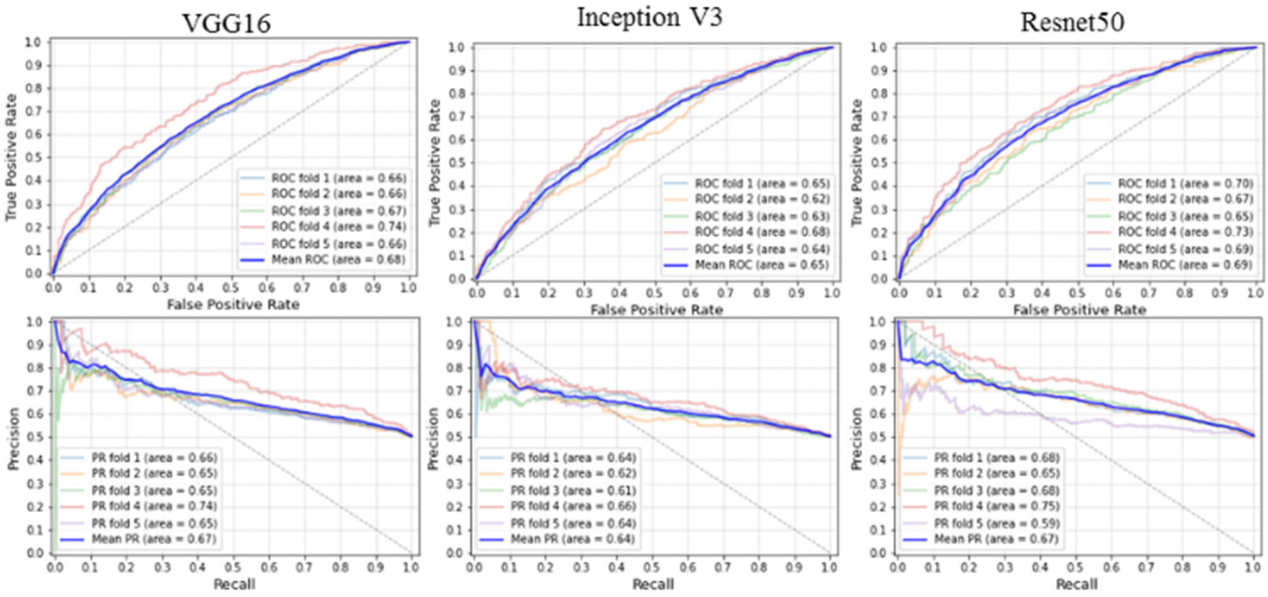

Figure S7. ROC and PR curves of female height predictions.
