# Supplementary material for: Application of Convolutional Neural Network for Fingerprint-Based Prediction of Gender, Finger Position, and Height
Source: Entropy (Basel). 2022 Mar 29;24(4):475. doi: 10.3390/e24040475 (PMC9029985; doi:10.3390/e24040475)
Supplement: Supplementary file 1 [file entropy-24-00475-s001.zip › Supplementary File S3.pdf]

Supplementary File S3. The predictions of each finger position

Table S1. Finger position prediction testing results obtained from five validated models of VGG16 (average accuracy of 84.8%)

True \ Predict	L1	L2	L3	L4	L5	R1	R2	R3	R4	R5	Accuracy (Mean±STD)
L1	193	3.2	1	0.2	0	1.2	1.2	0	0.2	0	96.5±0.7%
L2	1.2	164.6	16	1.4	3	0.2	3	1.4	2	7.2	82.3±3.0%
L3	3.6	12.6	158.6	12.2	4.2	0	0.6	2.2	5.4	0.6	79.3±2.2%
L4	0.2	1.6	11.4	150	20.8	0	5.8	5.2	4.4	0.6	75.0±3.0%
L5	0	0.8	1.4	17.4	172	0	6.8	0.8	0.6	0.2	86.0±2.9%
R1	1	0.4	0.6	0	0	191	3.4	3.6	0	0	95.5±2.4%
R2	0.2	1.8	0	1.8	3.6	0.8	175.8	11.2	1.8	3	87.9±3.8%
R3	0.4	0.4	0.8	5.6	1	4	13.8	163.6	7	3.4	81.8±3.8%
R4	0.8	2.2	4.6	6.6	0	1	1.8	14.2	150.4	18.4	75.2±3.4%
R5	0	5.2	0.4	0.2	1	0	1.2	1.8	13.4	176.8	88.4±2.3%

Table S2. Finger position prediction testing results obtained from five validated models of Inception-v3 (average accuracy of 77.9%)

True \ Predict	L1	L2	L3	L4	L5	R1	R2	R3	R4	R5	Accuracy (Mean±STD)
L1	188.4	2.4	4	0.6	0	2.8	1	0.2	0.4	0.2	94.2±2.0%
L2	1.8	139.8	21.4	4.4	5.8	0.6	6.6	1.8	9.4	8.4	69.9±4.1%
L3	5.6	17	135.8	19.8	4.8	0.4	1.4	2.4	11	1.8	67.9±5.0%
L4	1	2.4	14.6	143.8	20.8	0	5.2	4.8	6.8	0.6	71.9±3.7%
L5	0	1.4	5.2	23.4	161	0	6.4	1.6	0.2	0.8	80.5±3.4%
R1	3.8	0.6	0	0	0	187.2	3	4.6	0.8	0	93.6±1.7%
R2	0.4	2.6	1.8	5.6	8.2	1.8	153.4	19.6	3	3.6	76.7±5.4%
R3	0.4	1.2	1.6	9	0.8	4.4	20.4	143.6	12.8	5.8	71.8±4.5%
R4	0.8	4.4	4.8	6.6	0	2.2	2.2	13.2	145	20.8	72.5±5.0%
R5	0	7	1	0.2	1.2	0	1.6	6	22.8	160.2	80.1±4.5%

Table S3. Finger position prediction testing results obtained from five validated models of Resnet50 (average accuracy of 83.9%)

True \ Predict	L1	L2	L3	L4	L5	R1	R2	R3	R4	R5	Accuracy (Mean±STD)
L1	191.2	3.6	2	0.2	0	2	0.8	0	0.2	0	95.6±1.3%
L2	1.4	161.8	16.4	3.2	3.6	0.2	2.2	0.6	5.2	5.4	80.9±4.4%
L3	3.4	12.4	158.2	10.6	3.4	0	1.2	1	9.2	0.6	79.1±2.8%
L4	0.6	2.6	18.4	141.6	20.8	0	3.4	5	7	0.6	70.8±4.3%
L5	0	1.4	4.2	21.6	165.4	0	4.2	1.2	0.6	1.4	82.7±3.3%
R1	1.2	0.2	0.4	0	0	192.6	2.2	3	0.4	0	96.3±3.0%
R2	0.6	2.8	1	2.6	5.2	1.6	165.8	15	3.6	1.8	82.9±3.6%
R3	0	0.4	1.8	8.8	1	4.6	13.8	154.8	11.4	3.4	77.4±5.6%
R4	0.6	3.2	6	4.6	0.2	1.2	2.4	8.2	159	14.6	79.5±2.9%
R5	0	6.4	1.6	0.2	0.8	0	1.6	4	17	168.4	84.2±2.2%
