# Supplementary material for: Application of Convolutional Neural Network for Fingerprint-Based Prediction of Gender, Finger Position, and Height
Source: Entropy (Basel). 2022 Mar 29;24(4):475. doi: 10.3390/e24040475 (PMC9029985; doi:10.3390/e24040475)
Supplement: Supplementary file 1 [file entropy-24-00475-s001.zip › Supplementary File S4.pdf]

Supplementary File S4. The predictions of height from specific finger position

Table S1. Male height prediction testing results obtained from five validated models of VGG16

Male	Finger Position	Tall		Short		Accuracy (Mean±STD)	Accuracy
		Correct	Incorrect	Correct	Incorrect		
VGG16	All	131.8	68.2	123.8	76.2	63.9±4.1%	63.9%
	Exclude Thumb	93	67	113.4	46.6	64.5±2.6%	64.5%
	L1+R1	23.4	16.6	30	10	66.8±4.4%	66.8%
	L2+R2	27.6	12.4	24.4	15.6	65.0±3.6%	65.0%
	L3+R3	24.2	15.8	29	11	66.5±6.6%	66.5%
	L4+R4	28.8	11.2	27	13	69.8±4.4%	69.8%
	L5+R5	28.2	11.8	26	14	67.8±4.5%	67.8%

Table S2. Male height prediction testing results obtained from five validated models of Inception-v3

Male	Finger Position	Tall		Short		Accuracy (Mean±STD)	Accuracy
		Correct	Incorrect	Correct	Incorrect		
Inception V3	All	124.2	75.8	131	69	63.8±3.1%	63.8%
	Exclude Thumb	96.4	63.6	109.8	50.2	64.4±1.8%	64.4%
	L1+R1	20.2	19.8	26.6	13.4	58.5±6.8%	58.5%
	L2+R2	23.6	16.4	25	15	60.8±2.6%	60.8%
	L3+R3	28.2	11.8	21.8	18.2	62.5±4.4%	62.5%
	L4+R4	25.4	14.6	25.4	14.6	63.5±3.8%	63.5%
	L5+R5	23.6	16.4	25.4	14.6	61.3±2.7%	61.3%

Table S3. Male height prediction testing results obtained from five validated models of Resnet50

Male	Finger Position	Tall		Short		Accuracy (Mean±STD)	Accuracy
		Correct	Incorrect	Correct	Incorrect		
Resnet50	All	113	87	141.8	58.2	63.7±2.2%	63.7%
	Exclude Thumb	84.4	75.6	123.8	36.2	65.1±2.7%	65.1%
	L1+R1	25	15	24	16	61.3±2.8%	61.3%
	L2+R2	23.6	16.4	24.2	15.8	59.8±8.0%	59.8%
	L3+R3	19.4	20.6	30	10	61.8±5.8%	61.8%
	L4+R4	27.8	12.2	26	14	67.3±4.9%	67.3%
	L5+R5	28.4	11.6	22.4	17.6	63.5±2.4%	63.5%

Table S4. Female height prediction testing results obtained from five validated models of VGG16

Female	Finger Position	Tall		Short		Accuracy (Mean±STD)	Accuracy
		Correct	Incorrect	Correct	Incorrect		
VGG16	All	123.4	76.6	126.4	73.6	62.5±1.9%	62.5%
	Exclude Thumb	91.4	68.6	111.4	48.6	63.4±2.9%	63.4%
	L1+R1	23.6	16.4	26.6	13.4	62.8±7.3%	62.8%
	L2+R2	26.8	13.2	20.2	19.8	58.8±5.4%	58.8%
	L3+R3	16.4	23.6	32.4	7.6	61.0±6.8%	61.0%
	L4+R4	27.2	12.8	23	17	62.8±5.8%	62.8%
	L5+R5	23.8	16.2	29.8	10.2	67.0±4.2%	67.0%

Table S5. Female height prediction testing results obtained from five validated models of Inception-v3

Female	Finger Position	Tall		Short		Accuracy (Mean±STD)	Accuracy
		Correct	Incorrect	Correct	Incorrect		
Inception V3	All	146.8	53.2	95.2	104.8	60.5±2.3%	60.5%
	Exclude Thumb	101.6	58.4	96.2	63.8	61.8±2.2%	61.8%
	L1+R1	19.2	20.8	25.2	14.8	55.5±5.6%	55.5%
	L2+R2	18.8	21.2	28	12	58.5±4.5%	58.5%
	L3+R3	27	13	20.4	19.6	59.3±4.6%	59.3%
	L4+R4	27.8	12.2	18.6	21.4	58.0±5.0%	58.0%
	L5+R5	28.6	11.4	26.4	13.6	68.8±8.1%	68.8%

Table S6. Female height prediction testing results obtained from five validated models of Resnet50

Female	Finger Position	Tall		Short		Accuracy (Mean±STD)	Accuracy
		Correct	Incorrect	Correct	Incorrect		
Resnet50	All	98.4	101.6	152.8	47.2	62.8±3.6%	62.8%
	Exclude Thumb	85.2	74.8	123.8	36.2	65.3±3.7%	65.3%
	L1+R1	15.8	24.2	29.6	10.4	56.8±7.1%	56.8%
	L2+R2	21	19	26.2	13.8	59.0±6.5%	59.0%
	L3+R3	25	15	25.4	14.6	63.0±8.5%	63.0%
	L4+R4	21.8	18.2	27	13	61.0±7.6%	61.0%
	L5+R5	24.8	15.2	24.4	15.6	61.5±6.8%	61.5%
